# Solving the class imbalance problem using ensemble algorithm: application of screening for aortic dissection

**DOI:** 10.1186/s12911-022-01821-w

**Published:** 2022-03-28

**Authors:** Lijue Liu, Xiaoyu Wu, Shihao Li, Yi Li, Shiyang Tan, Yongping Bai

**Affiliations:** 1 School of Automation, Central South University, Changsha, 410083 Hunan China; 2 Hunan Zixing Artificial Intelligence Research Institute, Changsha, 410007 Hunan China; 3 Xiangya Hospital, Central South University, Changsha, 410008 Hunan China

**Keywords:** Class imbalance, Ensemble learning, SVM, Aortic dissection

## Abstract

**Background:**

Imbalance between positive and negative outcomes, a so-called class imbalance, is a problem generally found in medical data. Despite various studies, class imbalance has always been a difficult issue. The main objective of this study was to find an effective integrated approach to address the problems posed by class imbalance and to validate the method in an early screening model for a rare cardiovascular disease aortic dissection (AD).

**Methods:**

Different data-level methods, cost-sensitive learning, and the bagging method were combined to solve the problem of low sensitivity caused by the imbalance of two classes of data. First, feature selection was applied to select the most relevant features using statistical analysis, including significance test and logistic regression. Then, we assigned two different misclassification cost values for two classes, constructed weak classifiers based on the support vector machine (SVM) model, and integrated the weak classifiers with undersampling and bagging methods to build the final strong classifier. Due to the rarity of AD, the data imbalance was particularly prominent. Therefore, we applied our method to the construction of an early screening model for AD disease. Clinical data of 523,213 patients from the Institute of Hypertension, Xiangya Hospital, Central South University were used to verify the validity of this method. In these data, the sample ratio of AD patients to non-AD patients was 1:65, and each sample contained 71 features.

**Results:**

The proposed ensemble model achieved the highest sensitivity of 82.8%, with training time and specificity reaching 56.4 s and 71.9% respectively. Additionally, it obtained a small variance of sensitivity of 19.58 × 10^–3^ in the seven-fold cross validation experiment. The results outperformed the common ensemble algorithms of AdaBoost, EasyEnsemble, and Random Forest (RF) as well as the single machine learning (ML) methods of logistic regression, decision tree, k nearest neighbors (KNN), back propagation neural network (BP) and SVM. Among the five single ML algorithms, the SVM model after cost-sensitive learning method performed best with a sensitivity of 79.5% and a specificity of 73.4%.

**Conclusions:**

In this study, we demonstrate that the integration of feature selection, undersampling, cost-sensitive learning and bagging methods can overcome the challenge of class imbalance in a medical dataset and develop a practical screening model for AD, which could lead to a decision support for screening for AD at an early stage.

## Background

With the development of technology and digital medical data, computer techniques have been widely applied in the medical field. However, medical datasets are often imbalanced [1], for example, the non-patients/negative class set, has far more samples than the patients/positive class set. And the class imbalance problem is a typical problem in classification tasks [2]. When the dataset is imbalanced, in order to improve accuracy, many classifiers tend to misclassify minority samples into majority samples, even though a classifier that classifies all the samples into the majority class can get an accuracy of up to 98%. Obviously, the classifier is invalid because it cannot identify patients effectively. Therefore, accuracy is not an appropriate evaluation metric, and sensitivity and specificity are often used for evaluation in medical treatment instead. In particular, sensitivity always attracts more attention, which shows the ability of classifiers to find all positive samples. Misclassifying the patients class set leads to more serious consequences than misclassifying the non-patients class set.

There are three categories of strategies to solve the problem of class imbalance: the data-level approach, the algorithm-level approach and ensemble learning techniques [3, 4]. The data-level approach includes oversampling, undersampling and feature selection. Oversampling generates minority samples. Its disadvantage is that it causes overfitting and increases time complexity accordingly. Undersampling selects a part of the data from the majority set and recombines the minority set into a new dataset, which causes loss of information. Zhou et al. [5] and Feng et al. [6] revealed that combining sampling techniques and ensemble methods could solve the problem of information loss effectively. Feature selection based on the importance of factors can identify the most relevant factors for the classification. It can compress the dimensionality of the feature space. Because class imbalance problems are usually accompanied by high dimensionality of the data, it is important to adopt feature selection techniques. Researchers have shown it can alleviate the class imbalance problem to a certain extent [7].

The algorithm-level method mainly applies cost-sensitive learning methods [8], which are an extension of the weight adjustment method, by assigning higher weights to the minority class samples to modify their preference for the majority class. Many studies have demonstrated that ensemble learning techniques can achieve better performance than a single classifier when the dataset is imbalanced [9, 10]. Ensemble learning techniques combine multiple weak classifier models to obtain a better and more comprehensive strong model. There are two ways to integrate base classifiers into a strong classifier: bagging and boosting. The bagging method is a parallel ensemble techniques in which the base classifiers are generated in parallel, while the boosting method is a sequential method where the base classifiers are generated sequentially, with the later classifiers influenced by the earlier ones. The boosting method runs slowly and is sensitive to abnormal data and noise. In many real-world applications, one strategy cannot solve the class imbalance problem effectively. Usually several strategies are combined to solve the imbalance problem. Feng et al. [11] improved the performance of the general vector machine (GVM) by feature selection and cost-sensitive learning methods. Tao et al. [12] adopted cost-sensitive SVM and the boosting ensemble method for imbalanced dataset classification. Mustafa et al. [13] solved the class imbalance problem by combining undersampling techniques with the MultiBoost ensemble method. Seiffert et al. [14] showed that both sampling and the ensemble technique can improve the accuracy of skewed data streams effectively. Sainin et al. [15] applied feature selection and sampling methods to improve the ensemble model for the class imbalance problem.

Aortic dissection (AD) is a cardiovascular disease caused by the rupture of the aortic intima, in which the blood breaks through the aorta to form pathological changes in the true and false lumen. This is a very rare clinical emergency with low morbidity, a high rate of misdiagnosis and a high mortality rate [16]. And the number of non-patients is much larger than patients. It has been reported that the first 90 min in the early stage of AD is the prime time for treatment. In one study [17], the death rate was 21% for an AD patient untreated in the first 24 h, 37% for 48 h and 74% for one week. Most patients who are not treated will die within a year [18]. Current studies have limited understanding of the causes of AD. Although there are many known pathogenic factors for AD including family history of AD, pre-existing AD or aortic valve disease, hypertension, and cigarette smoking, [19], there is no highly sensitive and specific indicator [20]. At present, the golden criteria of AD diagnosis is CTA (computer tomography angiography) [21]. This check uses imaging detection to show the location, scope, entrance, exit and involvement of the aortic branches and aortic valve. Because AD has an insidious onset, primary medical institutions often face many difficulties in the diagnosis and prognosis of the disease. When facing a patient, the doctor will first inquire about the patient's medical history and physical examination results. Once the doctor feels the patient is at high-risk due to medical history and the presence of typical symptoms, CTA will be arranged to help confirm the diagnosis. The typical symptoms of AD are sudden severe pain in the chest, back and between the shoulder blades. However, some patients do not have typical symptoms. They may experience chest tightness, syncope, nausea and other symptoms, and these atypical symptoms are diverse. Many doctors lack the ability to distinguish and diagnose atypical AD patients, which leads them not to arrange a CTA. Thus, some patients with AD fail to get an accurate diagnosis and effective treatment in time.

Therefore, earlier screening and prediction of AD is essential. To help doctors screen for patients with suspected AD, doctors can take the screening results as advice and further examine those high-risk patients to then make an accurate diagnosis. Some researchers have used machine learning (ML) techniques to diagnose AD patients. Huo et al. [22] applied data mining methods including SVM, Naïve Bayes, Bayesian Network and J48 to classify AD patients, and the Bayesian network performed best with an accuracy of 84.55%. However, the purpose of their study was to identify false positive patients in 492 emergency cases who were sent to emergency room as AD patients. Their research is not suitable for early screening. Liu et al. [23] used multiple ensemble learning methods to screen for AD patients; however, they only explored the performance of existing ensemble methods.

In recent years, many ML approaches have been proposed for classification and medical treatment. Saadatfae et al. [24] proposed a new KNN algorithm that improved the pruning process of the LC-KNN. The results showed their method performed better than recent related works. Simon et al. [25] evaluated the performance of logistic regression and other ML algorithms to predict the risk of cardiovascular diseases and other diseases. Among them, logistic regression achieved as good of a performance as other ML models. A review [26] investigated the state-of-the-art research on deep learning techniques in the healthcare system between 2015 and 2019, which concluded that ensemble techniques based on deep learning techniques performed better than a single method. Ashish [27] applied SVM and the extreme gradient boosting method to detect ischemic heart disease using the Z-Alizadeh Sani dataset. Among various ML algorithms, SVM has proven to be one of the most outstanding methods [28]. The main idea of SVM [29] is to establish an optimal decision hyperplane to maximize the distance between the two types of samples closest to the plane, thereby providing good generalization for classification problems. However, SVM does not take into consideration the class distribution and class imbalance problem. In order to handle this problem, Veropoulos et al. [30] adjusted the loss function of SVM by modifying two different misclassification cost values. Kang et al. [31] proposed a weighted undersampling method for SVM; the improved algorithm performed well on imbalanced data sets. Hazarika [32] proposed a SVM that weights the training points based on their class distributions. Recently, the use of ensemble learning on SVM has been useful and has attracted much attention [33]. Pouriyeh et al. [34] investigated different ML methods for heart disease prediction. Then ensemble learning techniques, including stacking, bagging and boosting, were applied to optimize performance. The SVM method using the boosting approach performed best. Huang et al. [35] applied different ML methods to classify supraventricular ectopic and ventricular ectopic beats. The SVM ensemble method outperformed other methods. Shorewala et al. [36] compared the performance of base ML classifiers and their ensemble techniques in detecting coronary heart disease, and the stacking model involving SVM, RF and KNN performed best. Alsafi et al. [37] proposed a ML system to diagnose coronary heart disease. They integrated RF, SVM and XGBoost techniques to build a diagnosis model after feature selection and optimized oversampling on an unbalanced dataset.

In our work, we have explored the binary class imbalance problem in medical research, and tested our method in an early screening model for AD. The significant contributions are as follows:

1. An effective ensemble model, which integrates the bagging, data-level and algorithm-level methods, is proposed to overcome the class imbalance problem; it outperforms standard competitive base and ensemble classifiers.
2. Different data-level methods are used to deal with the class imbalance problem. First, feature selection techniques, including a significance test and logistic regression, are used for selecting relevant features. Then we integrate the weak classifiers with undersampling and bagging to build the final strong classifier.
3. The cost-sensitive learning method is applied to SVM models to construct weak classifiers by assigning higher misclassification cost to the minority class examples; this is different from the decision tree used by general ensemble models.
4. The proposed ensemble model is able to effectively identify patients with AD and also yields better results than the clinical screening results of some hospitals, indicating it can be used to develop a decision support for screening for AD at an early stage.

## Methods

**Fig. 1:**
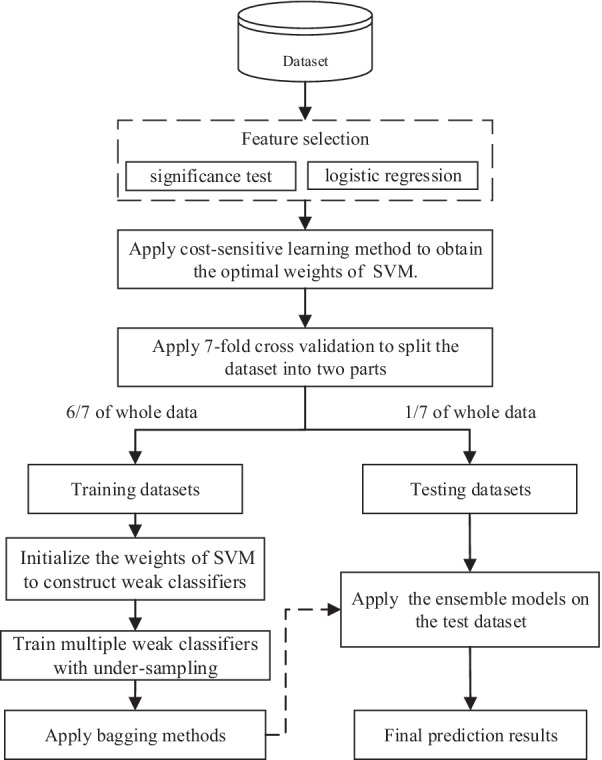
Data flow diagram of the proposed method

Our method consists of three parts: feature selection, cost-sensitive learning and the proposed ensemble algorithm. The three parts will be introduced in the following sections. The data flow diagram of the proposed method is shown in Fig. 1. The data-level method based on feature selection is applied to select the most relevant features by significance test and logistic regression methods. Then the algorithm-level method based on cost-sensitive learning is implemented on SVM by assigning different misclassification cost values for two classes to obtain the optimal weight settings $w$ of SVM. The seven-fold cross-validation technique is used to evaluate the predictive performance of the model. First, the dataset is partitioned into seven subsets evenly, and each subset is taken as a testing dataset. The remaining six subsets are used as the training dataset. In this way, seven models are obtained, and the average performance indicators of these models on the testing sets are used as the model’s final results.

During each training phase, the proposed ensemble algorithm was applied to obtain a better and more comprehensive ensemble model. The data-level method based on undersampling and ensemble learning techniques based on bagging were used. First, the weight settings $w$ are initialized on SVM to construct weak classifiers according to the results of cost-sensitive learning. Then multiple weak classifiers are trained using the balanced dataset obtained by undersampling. Finally, an ensemble model is constructed with weak classifiers by bagging.

During each testing phase, the result of the ensemble model on the testing dataset is predicted.

We compare the ensemble model to single classifiers, including logistic regression, KNN, decision tree, BP and SVM, as well as standard ensemble models including EasyEnsemble, AdaBoost and RF.

### Data collection

Since screening for AD patients is a typical imbalance problem, this study used an AD dataset. Clinical data of more than 60,000 cardiovascular in-patients were collected from the Institute of Hypertension, Xiangya Hospital, Central South University between 2008 and 2016. We referred to the indicators recommended in the 2014 ESC Guidelines and selected 71 features initially, including blood routine, biochemical examination, clotting routine examination and other easily accessible information, such as clinical presentation and medical history. The imbalance ratio of AD patients to non-AD patients is 1:65. Since any imbalance ratio more than 1:50 is considered a severe imbalance problem, predicting AD is such a problem. Details of these features are shown in Table 2. The use of all data was authorized by the Institute of Hypertension, Xiangya Hospital, Central South University.

**Fig. 2:**
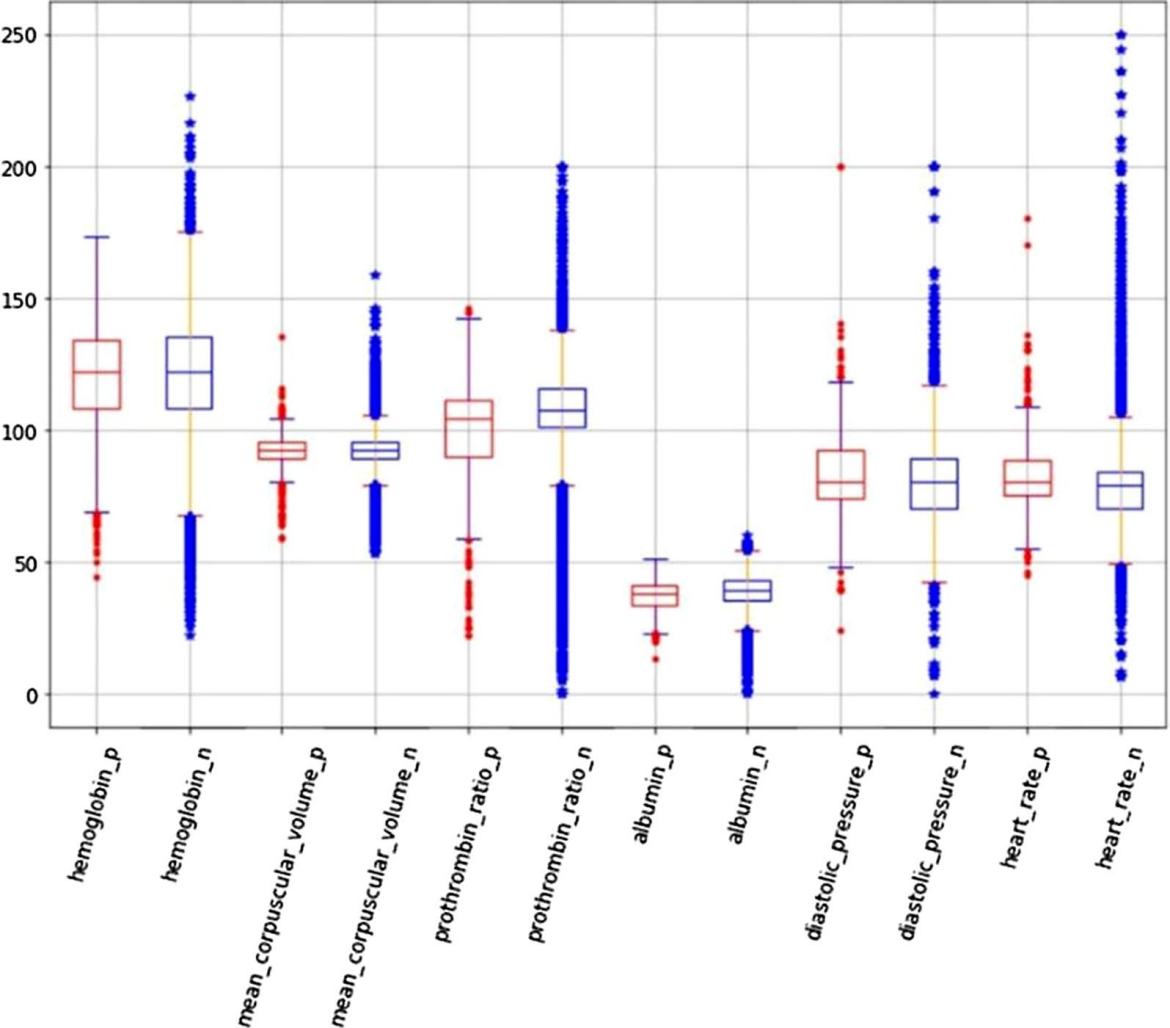
A box plot of randomly selected dataset features

**Fig. 3:**
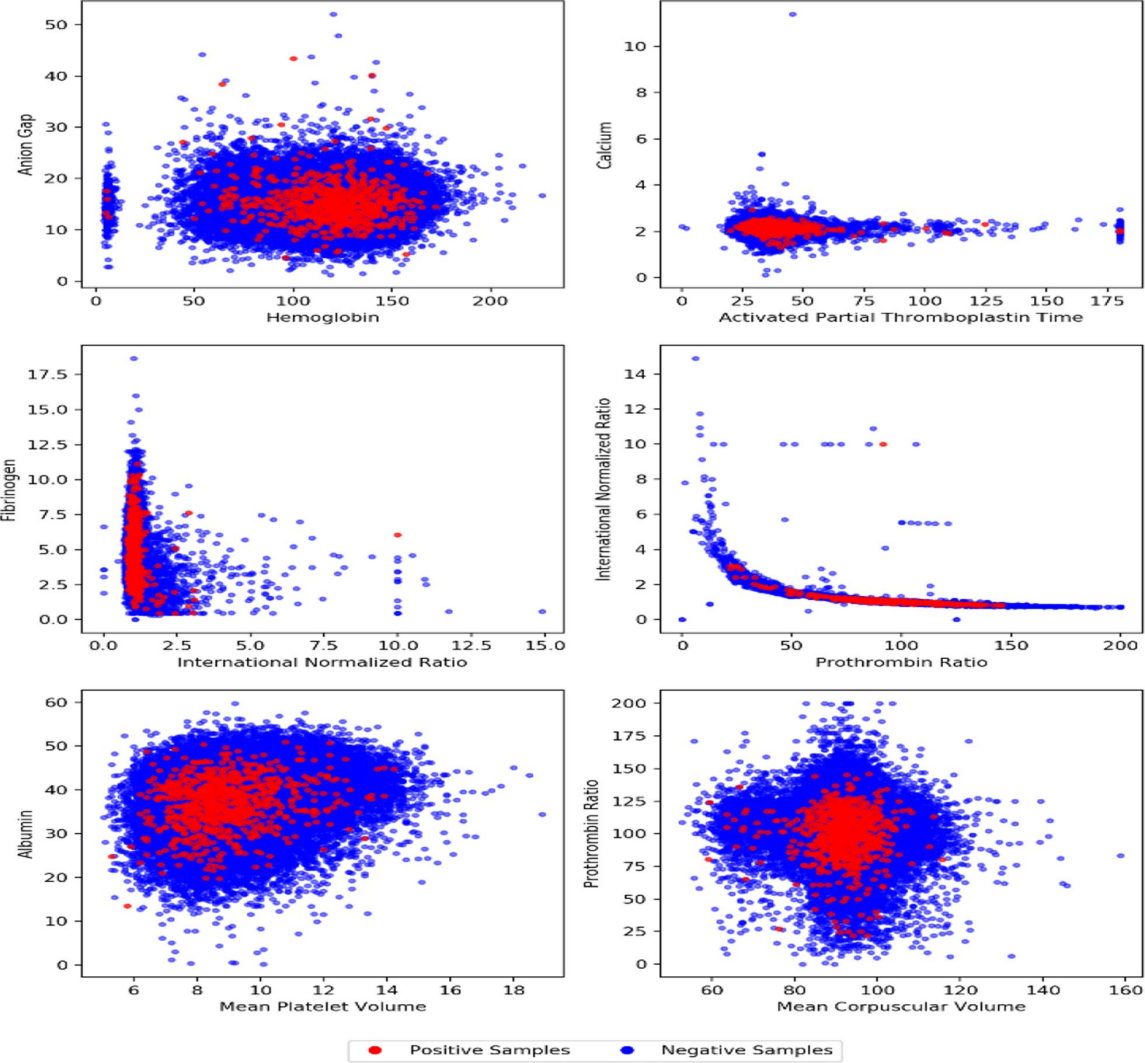
Scatter diagrams of dataset features

In order to have a comprehensive view of the data, box plots and scatter diagrams were drawn for every feature. The goal was to find some specific indicators that were helpful for classification but failed, which means it is difficult to distinguish an AD patient from non-patients using only one or a few indicators. Figure 2 is a box plot of some randomly selected features of our dataset. In a box plot, the horizontal line inside the box is the median value of the distribution. The upper and lower ends of the box are the approximate upper and lower quartiles of the distribution, and the whiskers extend 1.5 times the interquartile range (IQR) from the box edges. The box plot allows for identification of outliers in the distribution. The positive samples are drawn in red while the negative samples are blue in the box plot, which clearly shows that the distribution of positive samples is similar to that of negative samples; thus, it is difficult to separate positive and negative samples through a single feature. Figure 3 shows a set of scatter diagrams; each diagram is drawn using two different features of our dataset. From each individual diagram a serious overlap between positive and negative classes can be found, so it is also hard to separate positive samples from the negative with two features.

### Feature selection

Investigating the features that affect models can help to analyze the importance of them. Furthermore, feature selection techniques based on the importance of features play a crucial role in medical diagnosis and have been widely applied. They can reduce the dimensionality of features in data, and improve the performance of classifiers. Redundant features or poor features can make classifiers inaccurate. Aghaei et al. [38] analyzed factors associated with HIV-related stigma, and concluded strategies of diminishing the HIV-related stigma. Joloudari et al. [39] applied feature selection technology to improve the accuracy of coronary artery disease diagnosis. Four ML models were used to establish predictive models and select features, among which RF performed best. Liu et al. [40] proposed an embedded feature selection technology using a weighted Gini index on a decision tree for classification of imbalanced data. Singh et al. [41] determined relevant features for breast cancer prediction by significance analysis and feature selection methods. Ma et al. [42] studied eight feature selection techniques, and recursive feature elimination (RFE) based on SVM performed well. Huo et al. [22] applied the correlation-based feature selection (CFS) method to select attributes that were used to build ML models for AD classification. Wang et al. [43] investigated six filter-based feature selection techniques, such as information gain and chi-square [44]. Different ML classifiers and performance metrics were applied to build and evaluate models. Abdar [45] applied four ML classifiers, including decision tree, KNN, SVM and neural network to predict heart disease. Logistic regression was used to select significant variables.

In order to select relevant features, statistical analysis, including a significance test and logistic regression, were applied to analyze the influence of features.

A significance test is used to determine whether the difference between the experimental treatment group and the control group is statistically significant. In the significance test, categorical variables were presented as frequencies with percentages, and were analyzed by Chi-square test (${\upchi }^{2}$). Continuous variables were expressed as the mean with standard deviation (SD) and analyzed by independent t-tests. The P value less than 0.05 was considered to be statistically significant. Logistic regression is a type of regression analysis commonly used in the analysis of diseases. This method can analyze the relative importance of some factors in disease prediction. Therefore, we pinpointed the most relevant factors by using logistic regression.

Finally, the feature set $\mathrm{Fset}$ was constructed according to the following formula, including all features whose P values in ${\mathrm{F}}_{\mathrm{s}}$ and ${\mathrm{F}}_{\mathrm{l}}$ were no greater than 0.05.

$$\mathrm{Fset}={\mathrm{F}}_{\mathrm{s}}\cup {\mathrm{F}}_{\mathrm{l}},$$

where ${\mathrm{F}}_{\mathrm{s}}$ is the feature set selected by significance test; ${\mathrm{F}}_{\mathrm{l}}$ is the feature set selected by logistic regression.

In addition, feature selection based on RF and recursive feature elimination (RFE) were used to verify the effectiveness of the features selected in our study. RF is an ensemble learning method that uses multiple decision trees and has high accuracy and good robustness. It can quantify the importance of features through the attenuation of the Gini coefficient obtained by the decision tree. The main idea of RFE is to iteratively build a model to remove features. Then the process is repeated on the remaining features until all the features are traversed. The order of eliminating features in this process is the rank of feature importance. RFE is a greedy algorithm for finding the optimal feature subset. SVM model was used as the model of RFE in our study.

### Cost-sensitive learning

SVM is good at high dimension data, making it popular for many ML practitioners. Furthermore, in the SVM model, by changing the weights of positive and negative samples in the loss function, different penalty coefficients can be set for positive and negative samples, which means two different misclassification cost values will be assigned. For instance, the greater the weight of the positive sample, the greater the penalty for this type of sample, and the greater the penalty, the smaller the error it can tolerate. The loss function of SVM is the sum of the hinge loss function and the regularization term, which is computed as follows:

$$\sum_{i}^{N}{[1-{y}_{i}(w\bullet {x}_{i}+b)]}_{+}+\lambda {||w||}^{2},$$

where ${x}_{i}$ is the ${t}^{th}$ samples; ${y}_{i}$ is the class label of ${x}_{i}$; $w$ and b are the parameters of the hyperplane. ||*|| is the L2 norm. $If {x}_{i}\epsilon P, w={w}_{1}; else {x}_{i}\epsilon N, w={w}_{2}.$

Based on the advantages of SVM, SVM was selected as the base classifier for the ensemble model in this study. It is different from standard ensemble learning methods, such as AdaBoost and EasyEnsemble, which use decision tree as the base classifier. SVM models can pay more attention to positive samples and alleviate the impact of class imbalance.

### Proposed ensemble algorithm

**Fig. 4:**
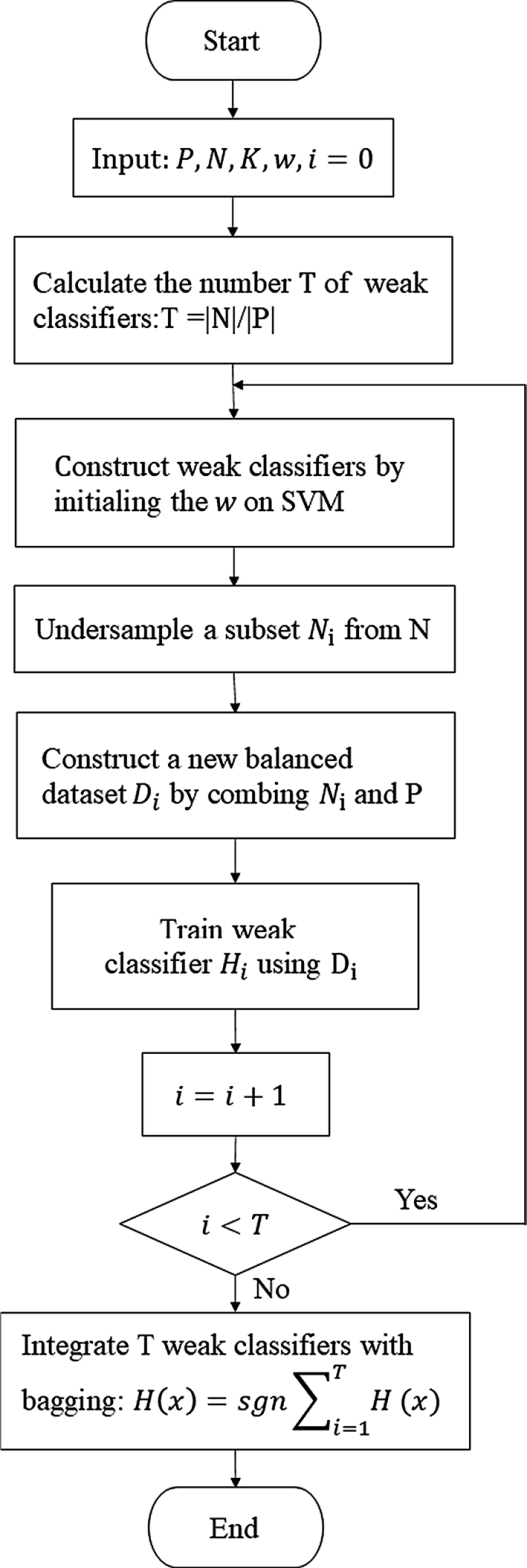
Flowchart of Algorithm 1

In our study, we focus on the binary class imbalance problem. The labels for the positive and negative samples were set to 1 and 0. The pseudo code of the proposed algorithm is shown in Algorithm 1, and the corresponding flowchart is shown in Fig. 4. The input of Algorithm 1 includes a dataset composed of a set of majority class samples $N$ and a set of minority class samples $P$, as well as K most relevant features obtained from feature selection, and the weight settings $w$ of SVM obtained from cost-sensitive learning. First calculate T, the number of weak classifiers based on the imbalanced ratio of major class set to minority class set. Then there is a loop to build and train T weak classifiers. In each loop, first construct the weak classifier ${H}_{i}(i=\mathrm{1,2},\dots ,T)$ by initializing the weight settings $w$ on SVM. Then randomly undersample a subset ${\mathrm{N}}_{i}(i=\mathrm{1,2},\dots ,\tau )$ from N and construct a new balanced dataset ${D}_{\mathrm{i}}$ by combining ${\mathrm{N}}_{i}$ and all instances of the minority class in P:

$$N={\bigcup }_{i=0}^{T}{N}_{i},$$

$${D}_{\mathrm{i}}={N}_{i}\cup P,$$

where $${N}_{i}\subset N; N=\bigcup_{i=1}^{T}{N}_{i}; {N}_{i}\cap {N}_{j}=\Phi \left(i\ne j\right); |{N}_{i}| = |P|$$.

Then train a weak classifier ${H}_{i}$ using ${D}_{\mathrm{i}}$. Repeat this process $T$ times until $T$ weak classifiers are all trained. Finally, an ensemble model $H\left(x\right)$ is built by integrating multiple weak classifiers with bagging methods.

### Performance evaluation

Usually, the performance of any classification algorithm is measured in terms of accuracy. However, relying only on classification accuracy, especially for an imbalanced medical dataset, could be misleading. Apparently, if a classifier identifies all the samples into the majority class, it can get a high accuracy. But this kind of classifier is meaningless. In this study, sensitivity and specificity were measured as two evaluation metrics as they are commonly used in the medical field. At the same time, training time was used as another metric to evaluate the complexity of the model. Sensitivity shows the ability to detect positive samples correctly to all positive samples. The higher the sensitivity, the lower the missed diagnosis rate. Specificity shows the ability to detect negative samples correctly to all negative samples. The higher the specificity, the lower the misdiagnosis rate. In the screening of diseases, it is more important to improve sensitivity, so as to reduce the missed diagnosis rate. Specificity does not need to be particularly high, and it is acceptable within a reasonable range. They are computed as follows:

$$\mathrm{specificity}= \frac{TN}{TN+FP},$$

$$\mathrm{sensitivity}= \frac{TP}{TP+FN},$$

where TP means the number of true positive samples; FN means the number of false negative samples; TN means the number of true negative samples, and FP means the number of false positive samples.

Each metric was tested under seven-fold cross validation that randomly selected six-sevenths of the dataset as the training set and one-seventh of the dataset as the test set. The undersampling method was employed to balance the training set.

## Results

### Data collection

After removing some samples with missing data, the dataset contains 53,213 samples. According to the hospital's discharge diagnosis records, among these samples, 802 cases are AD patients and 52,411 cases are non-patients. The imbalance ratio of positive samples to negative samples is 1:65. Among the 802 AD patients, there are 574 males (71.6%) and 228 females (28.4%); the age of the patients is between 18 and 89 years old, with an average of 55.57 ± 12.90, and 411 cases (51.2%) are between 50 and 70 years old. There are 618 (77.1%) drinkers, 506 (63.1%) smokers, and 596 (74.3%) suffer from chest pain.

### Experimental setup

Experiments were performed on a computer with 2.6 GHz CPU and 4 GB of RAM running Windows 7 as the operating system. Feature selection methods including logistic regression and a significance test were implemented using SPSS 25. Other feature selection and ML methods were performed using a Python 3.8 environment.

To get a better parameter for models in our study, a cross-validation grid search approach was used to search for the best parameter. The parameter of “n_estimators” of AdaBoost and Easy-Ensemble was set to 67, according to the imbalanced ratio of major class set to minority class set. Other unspecified parameters used the default settings. The model parameter settings used in our study are shown in Table 1.

**Table 1:** Experimental parameters of models

Models	Parameters
Logistic regression	C = 1, penalty = 'l2'
KNN	n_neighbors = 17
SVM	kernel = rbf, C = 4, degree = 3, gamma = 0.004
Decision tree	max_depth = 3
RF	n_estimators = 69
BP	hidden_layer_sizes = 142
AdaBoost	n_estimators = 65
Easy-Ensemble	n_estimators = 65
Proposed model	T = 65

### Feature selection

The significance test results are shown in Table 2. The serial numbers beginning with 1, 2, 3, and 4 indicate blood routine, biochemical examination, clotting routine examination and other indicators, respectively. Features with significant differences are shown in bold. There were 49 features in ${\mathrm{F}}_{\mathrm{s}}$ with a statistically significant difference (P < 0.05), including four indicators in blood routines, 17 in biochemical examination, seven in clotting routine examination and 21 in other.

**Table 2:** Significance test analysis of the indicators used to predict AD

Variables	AD N = 802		Non-AD N = 52,411		${\upchi }^{2}/\mathrm{t}$		P value
1.1 MCV	91.84 ± 6.82		92.10 ± 7.17		**− **1.04		0.161
**1.2 MPV**	**8.93 ± 1.39**		**9.3 ± 1.58**		**− 7.59**		** < 0.001**
1.3 HGB	119.76 ± 21.57		119.95 ± 22.47		**− **0.23		0.18
1.4 A/G	1.40 ± 0.36		1.49 ± 0.37		**− **6.71		0.88
**1.5 NEUT**	**7.16 ± 4.08**		**4.79 ± 3.47**		**19.12**		** < 0.001**
**1.6 NEUT%**	**72.83 ± 10.79**		**65.30 ± 12.09**		**17.53**		**0.02**
**1.7 LYMPH%**	**16.94 ± 9.10**		**24.22 ± 10.44**		**− 19.64**		**0.01**
1.8 LYMPH	1.36 ± 0.60		1.57 ± 2.03		**− **2.92		0.22
**2.1 TP**	**64.58 ± 7.06**		**65.43 ± 8.04**		**− 3.01**		** < 0.001**
**2.2 AIB**	**37.08 ± 5.67**		**38.61 ± 6.26**		**− 6.9**		**0.04**
2.3 GIOB	27.57 ± 5.19		26.94 ± 5.32		3.31		0.13
2.4 TB	16.19 ± 21.62		13.20 ± 26.81		3.13		0.07
2.5 DB	6.65 ± 11.52		5.39 ± 13.53		2.63		0.09
2.6 TBA	6.22 ± 13.32		7.55 ± 15.04		**− **2.49		0.17
**2.7 ALT**	**66.50 ± 296.27**		**32.47 ± 108.73**		**8.4**		** < 0.001**
**2.8 AST**	**85.34 ± 510.27**		**36.33 ± 155.39**		**8.28**		** < 0.001**
2.9 UREA	7.37 ± 5.21		7.06 ± 5.21		1.66		0.29
2.10 CREA	136.87 ± 156.07		138.98 ± 213.75		**− **0.28		0.08
**2.11 UA**	**337.62 ± 128.66**		**349.58 ± 116.65**		**− 2.88**		** < 0.001**
2.12 HBA1C	2.25 ± 0.62		2.03 ± 0.73		8.39		0.05
2.13 CHO	4.33 ± 0.43		4.37 ± 0.55		**− **2.19		0.81
2.14 HDL	1.12 ± 0.17		1.12 ± 0.17		**− **0.28		0.71
2.15 LDL	2.60 ± 0.35		2.63 ± 0.46		**− **1.98		0.51
**2.16 LDH**	**322.03 ± 684.10**		**236.51 ± 283.48**		**8.19**		** < 0.001**
**2.17 CK**	**538.04 ± 5272.64**		**162.57 ± 567.45**		**12.3**		** < 0.001**
**2.18 CK-MB**	**35.93 ± 299.32**		**19.33 ± 33.08**		**9.48**		** < 0.001**
**2.19 MYOG**	**72.69 ± 84.95**		**57.60 ± 59.02**		**7.13**		** < 0.001**
**2.20 K + **	**3.83 ± 0.56**		**3.97 ± 0.52**		**− 7.52**		** < 0.001**
**2.21 Na + **	**139.37 ± 4.28**		**140.71 ± 3.79**		**− 9.89**		** < 0.001**
**2.22 Cl-**	**101.08 ± 4.95**		**102.59 ± 4.62**		**− 9.15**		**0.01**
**2.23 CO2CP**	**23.14 ± 3.21**		**23.20 ± 3.65**		**− 0.45**		** < 0.001**
2.24 AG	15.24 ± 3.68		14.95 ± 3.35		2.38		0.7
**2.25 Ca + **	**2.16 ± 0.16**		**2.21 ± 0.18**		**− 7.85**		**0.01**
**2.26 P + **	**1.19 ± 0.39**		**1.19 ± 0.34**		**− 0.64**		**0.01**
2.27 Mg +	0.90 ± 0.13		0.89 ± 0.13		2.38		0.56
**2.28 ESR**	**31.34 ± 28.57**		**37.87 ± 30.34**		**− 6.054**		**0.023**
**2.29 FT3**	**3.77 ± 0.90**		**3.95 ± 1.60**		**− 3.32**		**0.034**
2.30 TSH	3.18** ± **7.86		3.52** ± **7.47		**− **1.258		0.442
**3.1 PT%**		**99.83 ± 18.59**		**106.62 ± 17.36**	**− 10.984**	** < 0.001**	
**3.2 INR**		**1.06 ± 0.39**		**1.01 ± 0.28**	**5.281**	** < 0.001**	
**3.3 APTT**		**37.66 ± 11.29**		**35.54 ± 9.68**	**6.14**	** < 0.001**	
**3.4 FIB**		**4.44 ± 1.81**		**3.77 ± 1.22**	**15.223**	** < 0.001**	
**3.5 D-Dimer**		**1.37 ± 1.94**		**0.97 ± 1.27**	**8.808**	** < 0.001**	
**3.6 PLGAg**		**252.01 ± 24.57**		**255.86 ± 27.68**	**− 3.914**	** < 0.001**	
3.7 TT		18.92 ± 14.17		19.11 ± 12.91	**− **0.398	0.078	
**3.8 PT**		**13.57 ± 4.39**		**13.02 ± 3.06**	**5.08**	** < 0.001**	
3.9 AT-III		271.19 ± 17.23		271.18 ± 21.52	0.01	0.77	
**4.1 Chest pain**		**206(25.69)**		**9460(18.05)**	**30.985**	** < 0.001**	
**4.2 Stomach ache**		**66(8.23)**		**2996(5.72)**	**9.199**	**0.002**	
**4.3 Heart palpitations**		**63(7.86)**		**6106(11.65)**	**11.099**	**0.001**	
**4.4 Dizziness and headache**		**62(7.73)**		**7803(14.89)**	**32.127**	** < 0.001**	
**4.5Aortic valve area murmur**		**23(2.87)**		**377(0.72)**	**48.875**	** < 0.001**	
**4.6 Family history of hypertension**		**92(11.47)**		**4798(9.15)**	**5.081**	**0.024**	
4.7 Family history of aortic dissection		0(0.00)		2(0.00)	0.031	0.861	
**4.8 Chest trauma history**		**11(1.37)**		**206(0.39)**	**18.623**	** < 0.001**	
**4.9 Hypertension**		**530(66.08)**		**31,571(60.24)**	**11.285**	**0.001**	
**4.10 Diabetes**		**88(10.97)**		**11,910(22.72)**	**62.467**	** < 0.001**	
**4.11 Family history of diabetes**		**8(1.00)**		**1480(2.82)**	**9.693**	**0.002**	
**4.12 Sex**		**228(28.43)**		**22,417(42.77)**	**66.471**	** < 0.001**	
**4.13 Hypertension and duration**		**6.01 ± 6.47**		**6.10 ± 7.09**	**− 0.33**	** < 0.001**	
**4.14 Smoking and duration**		**10.22 ± 14.39**		**7.34 ± 13.88**	**5.831**	** < 0.001**	
**4.15 Stop smoking and duration**		**0.57 ± 2.64**		**0.78 ± 3.26**	**− 1.82**	** < 0.001**	
**4.16 Drinking and duration**		**6.62 ± 11.68**		**5.57 ± 11.10**	**2.652**	** < 0.001**	
**4.17 Stop drinking and duration**		**0.17 ± 1.12**		**0.24 ± 1.65**	**− 1.209**	**0.016**	
**4.18 Systolic pressure**		**142.41 ± 26.71**		**136.86 ± 21.90**	**7.091**	** < 0.001**	
**4.19 Diastolic pressure**		**83.20 ± 16.59**		**80.46 ± 13.01**	**5.896**	** < 0.001**	
4.20 Heart rate		81.74 ± 13.87		78.73 ± 14.20	5.967	0.31	
4.21 Age		55.57 ± 12.90		62.56 ± 13.06	**− **15.034	0.319	
**4.22 Smoking**		**0.66 ± 0.53**		**0.82 ± 0.51**	**− 9.114**	** < 0.001**	
**4.23 Drinking**		**0.81 ± 0.48**		**0.85 ± 0.44**	**− 2.426**	** < 0.001**	
**4.24 Diabetes and duration**		**0.85 ± 2.87**		**1.82 ± 3.83**	**− 7.113**	** < 0.001**	

The bold items were features selected by significance test

The underlined items were features selected by logistic regression and not by significance test

The logistic regression results are shown in Table 3. Variables which are significantly correlated with the target variable (P < 0.05) are in bold. There were 35 features in ${\mathrm{F}}_{1}$, including three indicators in blood routines, 12 in biochemical examination, four in clotting routine examination and 16 in other.

**Table 3:** Logistic regression analysis of the indicators used to predict AD

Variable	B	OR	95% CI	P value
1.1 MCV	0.009	1.009	(0.998–1.020)	0.129
**1.2 MPV**	**− 0.094**	**0.91**	**(0.863–0.960)**	** < 0.001**
1.3 HGB	**− **0.002	0.998	(0.994–1.002)	0.316
1.4 A/G	**− **0.444	0.642	(0.332–1.240)	0.187
1.5 NEUT	0.006	1.006	(0.989–1.022)	0.497
**1.6 NEUT%**	**− 0.018**	**0.982**	**(0.970–0.995)**	**0.005**
**1.7 LYMPH%**	**− 0.076**	**0.926**	**(0.910–0.943)**	** < 0.001**
1.8 LYMPH	**− **0.011	0.989	(0.920–1.063)	0.77
2.1 TP	0.044	1.045	(0.985–1.109)	0.145
2.2 AIB	**− **0.001	0.999	(0.935–1.067)	0.975
2.3 GIOB	**− **0.047	0.954	(0.892–1.021)	0.173
2.4 TB	0.012	1.012	(1.000–1.024)	0.053
2.5 DB	**− **0.014	0.986	(0.962–1.010)	0.257
**2.6 TBA**	**− 0.023**	**0.978**	**(0.968–0.988)**	** < 0.001**
2.7 ALT	0	1	(1.000–1.001)	0.306
2.8 AST	0	1	(1.000–1.000)	0.475
**2.9 UREA**	**0.027**	**1.028**	**(1.004–1.052)**	**0.022**
**2.10 CREA**	**− 0.002**	**0.998**	**(0.997–0.998)**	** < 0.001**
**2.11 UA**	**− 0.001**	**0.999**	**(0.998–1.000)**	**0.004**
**2.12 HBA1C**	**0.327**	**1.387**	**(1.253–1.535)**	** < 0.001**
2.13 CHO	**− **0.29	0.749	(0.523–1.072)	0.114
**2.14 HDL**	**0.69**	**1.994**	**(1.198–3.319)**	**0.008**
2.15 LDL	0.167	1.182	(0.787–1.774)	0.421
2.16 LDH	0	1	(1.000–1.000)	0.702
2.17 CK	0	1	(1.000–1.000)	0.189
2.18 CK-MB	0	1	(0.999–1.001)	0.687
2.19 MYOG	0.001	1.001	(1.000–1.002)	0.088
**2.20 K + **	**− 0.469**	**0.626**	**(0.535–0.732)**	** < 0.001**
2.21 Na +	**− **0.033	0.967	(0.919–1.019)	0.208
2.22 Cl-	0.001	1.001	(0.953–1.052)	0.956
2.23 CO2CP	0.03	1.03	(0.977–1.086)	0.273
2.24 AG	**− **0.002	0.998	(0.947–1.052)	0.94
**2.25 Ca + **	**− 0.656**	**0.519**	**(0.307–0.877)**	**0.014**
**2.26 P + **	**0.246**	**1.278**	**(1.007–1.622)**	**0.043**
**2.27 Mg + **	**0.552**	**1.737**	**(1.205–2.503)**	**0.003**
**2.28 ESR**	**− 0.008**	**0.992**	**(0.989–0.995)**	** < 0.001**
**2.29 FT3**	**− 0.153**	**0.858**	**(0.800–0.919)**	** < 0.001**
2.30 TSH	**− **0.009	0.991	(0.980–1.003)	0.154
**3.1 PT%**	**− 0.017**	**0.983**	**(0.977–0.989)**	** < 0.001**
3.2 INR	**− **0.197	0.821	(0.337–2.002)	0.665
3.3 APTT	0	1	(0.991–1.009)	0.96
3.4 FIB	**0.185**	**1.203**	**(1.145–1.265)**	** < 0.001**
**3.5 D-Dimer**	**0.056**	**1.058**	**(1.029–1.087)**	** < 0.001**
**3.6 PLGAg**	**− 0.006**	**0.994**	**(0.990–0.998)**	**0.002**
**3.7 TT**	**− 0.007**	**0.993**	**(0.986–1)**	**0.042**
3.8 PT	**− **0.005	0.995	(0.913–1.084)	0.902
3.9 AT-III	0.002	1.002	(0.999–1.006)	0.214
**4.1 Chest pain**	**0.643**	**1.903**	**(1.594–2.271)**	** < 0.001**
4.2 Stomach ache	0.084	1.088	(0.826–1.433)	0.55
4.3 Heart palpitations	**− **0.257	0.774	(0.588–1.017)	0.066
**4.4 Dizziness and headache**	**− 0.79**	**0.454**	**(0.347–0.594)**	** < 0.001**
**4.5 Aortic valve area murmur**	**1.563**	**4.774**	**(2.965–7.685)**	** < 0.001**
4.6 Family history of hypertension	0.07	1.073	(0.846–1.36)	0.56
4.7 Family history of aortic dissection	**− **16.477	0	0	1
**4.8 Chest trauma history**	**0.948**	**2.581**	**(1.336–4.985)**	**0.005**
**4.9 Hypertension**	**0.501**	**1.651**	**(1.336–2.040)**	** < 0.001**
**4.10 Diabetes**	**− 0.776**	**0.46**	**(0.364–0.582)**	** < 0.001**
**4.11 Family history of diabetes**	**− 0.909**	**0.403**	**(0.196–0.828)**	**0.013**
**4.12 Sex**	**− 0.529**	**0.589**	**(0.485–0.716)**	** < 0.001**
4.13 Hypertension and duration	**− **0.006	0.994	(0.978–1.009)	0.424
4.14 Smoking and duration	**− **0.002	0.998	(0.991–1.006)	0.679
4.15 Stop smoking and duration	**− **0.005	0.995	(0.966–1.026)	0.759
4.16 Drinking and duration	0.004	1.004	(0.994–1.015)	0.41
**4.17 Stop drinking and duration**	**− 0.096**	**0.908**	**(0.829–0.995)**	**0.039**
**4.18 Systolic pressure**	**0.018**	**1.018**	**(1.014–1.022)**	** < 0.001**
**4.19 Diastolic pressure**	**− 0.012**	**0.988**	**(0.981–0.995)**	**0.001**
**4.20 Heart rate**	**− 0.006**	**0.994**	**(0.989–0.999)**	**0.021**
**4.21 Age**	**− 0.054**	**0.948**	**(0.942–0.953)**	** < 0.001**
**4.22 Smoking**	**− 0.429**	**0.651**	**(0.537–0.790)**	** < 0.001**
**4.23 Drinking**	**0.35**	**1.419**	**(1.086–1.855)**	**0.01**
**4.24 Diabetes and duration**	**− 0.098**	**0.907**	**(0.882–0.932)**	** < 0.001**

B, unstandardized regression weight; OR, odds ratio; CI, confidence interval

The bold items were features selected by logistic regression

In summary, 26 features are in both ${\mathrm{F}}_{\mathrm{s}}$ and ${\mathrm{F}}_{\mathrm{l}}$; 23 features are only in ${\mathrm{F}}_{\mathrm{s}}$; nine features are only in ${\mathrm{F}}_{\mathrm{l}}$. Finally, the union of ${\mathrm{F}}_{\mathrm{l}}$ and ${\mathrm{F}}_{\mathrm{s}}$ was selected as the feature set, called Fset. There are 58 features in Fset to build prediction models, as listed in Table 2. The bold items are features in ${\mathrm{F}}_{\mathrm{s}}$. The underlined items are features in ${\mathrm{F}}_{\mathrm{l}}$ and not in ${\mathrm{F}}_{\mathrm{s}}$. Among the features in Fset, four indicators came from blood routines, 23 from biochemical examination, eight from clotting routine examination and 23 from other.

RF and RFE methods were used to rank the features according to their importance from the most important to the least important. According to statistical analysis, more than 90% of the top 58 features of the two methods are in the feature set selected in our study, indicating that the features selected are meaningful. Table 4 lists the top 10 common features of the two feature selection methods and their importance based on RF.

**Table 4:** Feature importance ranking

Features	Importance
2.17 CK	2.74%
3.6 PLGAg	2.68%
4.21 Age	2.63%
2.19 MYOG	2.53%
3.7 TT	2.37%
4.18 Systolic pressure	2.36%
3.4 FIB	2.30%
2.20K +	2.16%
2.28 ESR	2.14%
1.6 NEUT%	2.03%

### Cost-sensitive learning

In this study, the patients set is called the positive/minority class set, and the non-patients set is called the negative/majority class set. By adjusting the weight parameters in the loss function of the SVM model, we can reduce class imbalance by assigning higher weights to the minority class examples and making the model pay more attention to minority samples. In order to find the best combination of weights, we implemented cost-sensitive analysis on the weights, and compared the SVM models with different weight settings.

The results are shown in Table 5. In order to have more reliable and valuable test results, seven-fold cross-validation was used. The average row shows the average of the seven-fold cross validation results. In Table 5, SVM (1.3, 1) means that ${\mathrm{w}}_{1}=1.3$ and ${\mathrm{w}}_{2}=1$; ${\mathrm{w}}_{1}$ was the weight of the positive samples, and ${\mathrm{w}}_{2}$ was the weight of the negative samples. When the weight of the positive samples reaches 2, the specificity is too low. Therefore, SVM models with a weight greater than 2 on the positive samples were not considered.

**Table 5:** Sensitivity (Se) and specificity (Sp) of SVM models with different weights on positive and negative samples

	SVM (1,1)		SVM (1.3,1)		SVM (1.6,1)		SVM (2,1)	
	Se	Sp	Se	Sp	Se	Sp	Se	Sp
1st	0.772	0.792	0.825	0.746	0.842	0.697	0.868	0.653
2nd	0.746	0.807	0.754	0.751	0.754	0.691	0.789	0.669
3rd	0.781	0.768	0.816	0.727	0.860	0.675	0.868	0.644
4th	0.746	0.790	0.781	0.751	0.807	0.696	0.816	0.666
5th	0.772	0.805	0.781	0.756	0.798	0.684	0.851	0.646
6th	0.728	0.795	0.763	0.741	0.781	0.687	0.833	0.648
7th	0.771	0.779	0.847	0.727	0.873	0.679	0.898	0.642
Average	0.759	0.791	0.795	0.734	0.816	0.687	0.846	0.653

By changing the weights and sacrificing specificity slightly, a SVM model can be generated with higher sensitivity. The larger the weight of the positive samples, the higher the cost the model pays when it mistakenly assigns a positive class sample to a negative class; thus, our models focus more on positive samples. Such models are of great significance due to the fact that higher sensitivity may make the model less likely to miss a patient. The sacrifice of specificity is worthy to some extent because as an early warning system, our purpose is only to allow patients who receive an alert to undergo further examination to confirm the diagnosis. However, the specificity should not be too low because a model with specificity that is too low can lead to much wasted cost by healthy people who pay for unnecessary further examination. In this regard, SVM (1.3,1) is considered to be the best base model since it pursues a higher sensitivity and does not have a specificity that is too low.

### Performance of proposed ensemble model

According to the results of the sensitivity analysis, SVM (1.3, 1) performs better, so weak classifiers were constructed based on the SVM (1.3, 1).The ensemble model was built by multiple weak classifiers. Table 6 compares the training times of the three ensemble learning models. Table 7 compares their sensitivities and specificities. Table 8 compares the sensitivities and specificities of the base ML algorithms. To minimize errors, average values of the seven-fold cross validation were used as results, which were then used to explain the generalization abilities of the different models. A smaller value means more stable grades on different training sets; in other words, a stronger generalization ability.

**Table 6:** Training time of different models (unit: s)

	AdaBoost	EasyEnsemble	Ensemble model	Random Forest
1st	3.4	185.3	55.0	0.36
2nd	4.2	191.2	58.4	0.31
3rd	4.0	191.2	54.8	0.31
4th	3.6	188.2	56.0	0.32
5th	3.7	191.2	57.6	0.39
6th	3.8	179.4	55.2	0.39
7th	4.7	185.3	58.1	0.31
Average	3.9	187.3	56.4	0.34

**Table 7:** Sensitivity (Se) and specificity (Sp) of ensemble learning models

	AdaBoost		EasyEnsemble		Ensemble model		Random forest	
	Se	Sp	Se	Sp	Se	Sp	Se	Sp
1st	0.736	0.742	0.798	0.802	0.816	0.705	0.781	0.791
2nd	0.675	0.759	0.737	0.794	0.798	0.733	0.737	0.792
3rd	0.772	0.744	0.825	0.793	0.842	0.704	0.807	0.775
4th	0.631	0.765	0.702	0.816	0.807	0.724	0.693	0.789
5th	0.754	0.748	0.798	0.803	0.860	0.717	0.754	0.821
6th	0.631	0.762	0.781	0.802	0.825	0.730	0.728	0.810
7th	0.711	0.765	0.693	0.818	0.847	0.715	0.695	0.810
Average	70.1%	75.5%	76.1%	80.4%	82.8%	71.9%	74.2%	79.8%
Variance ($\times {10}^{-3}$)	57.23	10.3	51.49	9.75	19.58	9.89	42.27	15.79

**Table 8:** Sensitivity (Se) and specificity (Sp) of logistic regression, decision tree, KNN and BP

	Logistic regression		Decision tree		KNN		BP	
	Se	Sp	Se	Sp	Se	Sp	Se	Sp
1st	0.789	0.771	0.702	0.690	0.728	0.715	0.737	0.760
2nd	0.754	0.786	0.596	0.660	0.684	0.709	0.754	0.773
3rd	0.798	0.754	0.684	0.653	0.711	0.700	0.789	0.736
4th	0.711	0.783	0.658	0.680	0.789	0.693	0.746	0.749
5th	0.789	0.774	0.702	0.682	0.772	0.656	0.781	0.765
6th	0.754	0.774	0.667	0.667	0.667	0.679	0.711	0.765
7th	0.788	0.773	0.644	0.679	0.720	0.683	0.788	0.737
Average	0.769	0.774	0.665	0.673	0.724	0.691	0.758	0.755

**Fig. 5:**
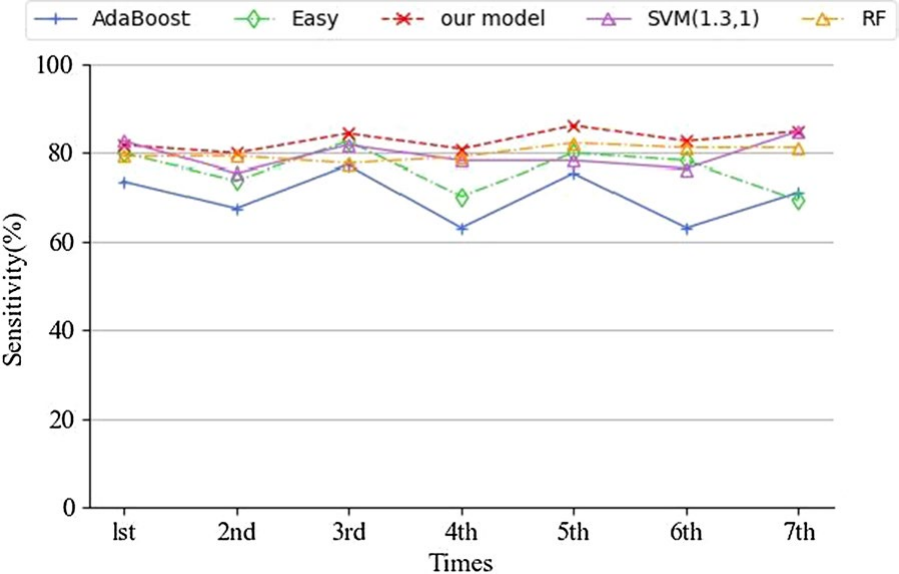
Seven-fold cross validation results of sensitivity of AdaBoost, EasyEnsemble (Easy), RF, Ensemble model and SVM (1.3, 1)

The model proposed in this article is named Ensemble model in the results. As can be seen from Table 6, RF achieved the lowest training time, followed by AdaBoost. The training time of EasyEnsemble is generally 50 times as much as AdaBoost. The training time of the Ensemble model is much shorter than that of EasyEnsemble. As can be seen from Table 7, the Ensemble model obtained a higher sensitivity (82.8%) than that of SVM (1.3, 1) (79.5%), although it had a lower specificity (71.9%). This is acceptable, because we pay more attention to improving sensitivity. Among the four ensemble learning models, AdaBoost performed poorly, while the Ensemble model performed the best. It achieved the highest sensitivity, and its specificity is still higher than 70%, which many routine diagnoses cannot reach. Moreover, the variance of the Ensemble model is obviously smaller than that of the other models, which means when dealing with different data sets, its performance will be relatively stable; in other words, it has a stronger generalization ability. This point is demonstrated more vividly by the fourth and seventh experiments, where when AdaBoost, EasyEnsemble and RF perform terribly on sensitivity, the Ensemble model does not perform too badly. It can be seen from Fig. 5 that the sensitivity of the Ensemble model is optimal and the most stable. Compared with the results of the base ML methods in Table 8, the ensemble methods demonstrated superior results. And logistic regression achieved a good performance with a sensitivity of 76.9% and a specifity of 77.4%, followed by BP.

## Discussion

Nowadays with the rapid growth of electronic medical data, greater challenges are presented to the issues of class imbalance. A recent review [46] showed that the problem of class imbalance in data mining is still common. The solutions to the problem of class imbalance are characterized by data-level, algorithm-level and ensemble learning techniques. Many researchers have explored solutions to imbalance problems. Undersampling, which divides the negative class sets and chooses only parts of them to participate in training a model, is commonly used to solve imbalance problems [47]. However, its deficiency is that it ignores many potentially useful major class examples. One previous study [48] indicated that the combination of ensemble methods and undersampling techniques could solve this problem effectively. In addition, Zhou et al. [5] revealed that the integration of ensemble methods and undersampling techniques kept the efficiency of undersampling. Gu et.al [49] proposed a fuzzy SVM for the class imbalance problem which is a modified class of SVM classifier with cost-sensitive methods that adjusts for the misclassification costs for two classes. Sainin et al. [15] applied feature selection and sampling methods to improve the ensemble model for the class imbalance problem, which combines the data-level method and ensemble methods. Velusamy et.al [50] combined three base classifiers to generate an ensemble method with a reduced feature subset on balanced datasets using the Synthetic Minority Over-sampling Technique (SMOTE). Many researchers have only combined the data-level method or algorithm-method with the ensemble learning techniques; however, there are few studies that combine three methods.

Due to the rarity of AD, the dataset we used is highly imbalanced, with high imbalance ratio of 65:1. Therefore, screening for AD is a significant imbalance problem. Hence, we applied the proposed ensemble method to the construction of an early screening model for AD disease to validate our model. AD is a cardiovascular emergency with low morbidity and high mortality. Due to its acute onset and complex clinical presentations, the rate of missed diagnosis and misdiagnosis is high [51]. Therefore, early screening of AD can effectively prevent later health loss and provide doctors with decision support. In recent years, some studies have applied ML techniques in AD diagnosis. Harris et al. [52] applied a convolutional neural network to classify AD and rupture on post-contrast CT images. Wu et al. [53] established a RF model to predict in-hospital rupture of type A AD using imaging examinations, clinical manifestations and other attributes of 1,133 patients. But these researchers focused on diagnosis, not screening. In the literature [23], four ML models have been used to screen for AD cases from imbalanced data, and SmoteBagging achieved the highest sensitivity of 78.1%. However, the complexity of this method was very high, requiring substantial computing resources, and the training time was more than 1000 s.

In the current study, an integrated learning approach combined data-level methods, algorithm-level methods and bagging ensemble techniques to address the problems posed by class imbalance. Class imbalance issues always lead to low sensitivity, which shows the ability of the classifier to find all patients. Since identifying patients is more important than identifying healthy people, the main objective in medical research with imbalanced datasets is to improve sensitivity. The experimental results show the sensitivity and specificity of the three ensemble models are over 70%, which is an obvious advantage over routine diagnostics [51, 54, 55], whose missed rate is between 35 and 45%. In other words, routine diagnostics, including the examination of CT and MR angiography, failed to identify many people who did suffer from AD, while others who did not get sick received unnecessary intervention. The ensemble model established in this study performed significantly better on sensitivity compared to other models. At the same time, our model has a lower complexity with a training time of 56.4 s. Additionally, the variance of the seven-fold cross validation was small, indicating that the model had stronger stability and generalization ability. In future work, we will investigate our method with different class imbalance ratios and datasets.

## Conclusion

We have presented a study on class imbalance classification using an AD dataset. We have demonstrated that the proposed ensemble model using bagging methods has great performance by combining feature selection, undersampling and cost-sensitive leaning on SVM. The ensemble model performed better than base classifiers and common ensemble learning algorithms with its highest sensitivity being 82.8%, which can find more positive outcomes. In healthcare research, class imbalance is a common phenomenon; the population of sick people is obviously less than non-sick people. Research in this area helps to provide an effective method to overcome the class imbalance problem.

## Data Availability

The datasets generated and/or analysed during the current study are not publicly available due to limitations of ethical approval involving the patient data and anonymity but are available from the corresponding author on reasonable request.

## Abbreviations

AD: Aortic dissection
CTA: Computer tomography angiography
ML: Machine learning
SVM: Support vector machine
KNN: K nearest neighbors
RF: Randoem forest
RFE: Recursive feature elimination
BP: Back propagation neural network

## References

[1] Belarouci S, Chikh MA (2017). Medical imbalanced data classification. Adv Sci Technol Eng Syst J..

[2] Bi J, Zhang C (2018). An empirical comparison on state-of-the-art multi-class imbalance learning algorithms and a new diversified ensemble learning scheme. Knowl Based Syst.

[3] Wu J, Zhao Z, Sun C, Yan R, Chen X. Learning from class-imbalanced data with a model-agnostic framework for machine intelligent diagnosis. Reliab Eng Syst Saf. 2021:107934.

[4] Liu X-Y. An empirical study of boosting methods on severely imbalanced data. In: International conference on advances in materials science and information technologies in industry (AMSITI); 2014; Xian, Peoples R China.

[5] Liu XY, Wu J, Zhou ZH (2009). Exploratory undersampling for class-imbalance learning. IEEE Trans Syst Man Cybern.

[6] Feng W, Huang W, Ren J. Class imbalance ensemble learning based on the margin theory. Appl Sci. 2018;8(5).

[7] Longadge R, Dongre SJIJoCS, Network. Class imbalance problem in data mining review. 2013;2(1).

[8] Zhou ZH, Liu XY (2006). Training cost-sensitive neural networks with methods addressing the class imbalance problem. IEEE Trans Knowl Data Eng.

[9] Hosni M, Abnane I, Idri A, Carrillo de Gea JM, Fernandez Aleman JL (2019). Reviewing ensemble classification methods in breast cancer. Comput Meth Programs Biomed.

[10] Khoshgoftaar TM, Van Hulse J, Napolitano A (2011). Comparing boosting and bagging techniques with noisy and imbalanced data. IEEE Trans Syst Man Cybern A Syst Hum.

[11] Feng F, Li KC, Shen J, Zhou Q, Yang X (2020). Using cost-sensitive learning and feature selection algorithms to improve the performance of imbalanced classification. IEEE Access..

[12] Tao X, Li Q, Guo W, Ren C, Li C, Liu R (2019). Self-adaptive cost weights-based support vector machine cost-sensitive ensemble for imbalanced data classification. Inf Sci.

[13] Mustafa G, Niu Z, Yousif A, Tarus J. Solving the class imbalance problems using RUSMultiBoost ensemble. In: 2015 10th Iberian conference on information systems and technologies (CISTI); 2015 17–20 June 2015.

[14] Seiffert C, Khoshgoftaar TM, Van Hulse J, Napolitano A (2010). RUSBoost: a hybrid approach to alleviating class imbalance. IEEE Trans Syst Man Cybern A Syst Humans..

[15] Sainin MS, Alfred R, Ahmad F (2021). Ensemble meta classifier with sampling and feature selection for data with imbalance multiclass problem. J Inf Commun Technol.

[16] Canaud L, Patterson BO, Peach G, Hinchliffe R, Loftus I, Thompson MM (2013). Systematic review of outcomes of combined proximal stent grafting with distal bare stenting for management of aortic dissection. J Thorac Cardiov Surg..

[17] Group JJW (2013). Guidelines for diagnosis and treatment of aortic aneurysm and aortic dissection (JCS 2011): digest version. Circ J.

[18] Crawford ES (1990). The diagnosis and management of aortic dissection. JAMA.

[19] Erbel R, Aboyans V, Boileau C, Bossone E, Di Bartolomeo R, Eggebrecht H (2014). 2014 ESC Guidelines on the diagnosis and treatment of aortic diseases. Eur Heart J.

[20] Erbel R, Alfonso F, Boileau C, Dirsch O, Eber B, Haverich A (2001). Diagnosis and management of aortic dissection - recommendations of the task force on aortic dissection, European Society of Cardiology. Eur Heart J.

[21] Vardhanabhuti V, Nicol E, Morgan-Hughes G, Roobottom CA, Roditi G, Hamilton MCK (2016). Recommendations for accurate CT diagnosis of suspected acute aortic syndrome (AAS)–on behalf of the British Society of Cardiovascular Imaging (BSCI)/British Society of Cardiovascular CT (BSCCT). Br J Radiol.

[22] Huo D, Kou B, Zhou Z, Lv M (2019). A machine learning model to classify aortic dissection patients in the early diagnosis phase. Sci Rep.

[23] Liu LJ, Zhang CW, Zhang GG, Gao Y, Luo JM, Zhang W (2020). A study of aortic dissection screening method based on multiple machine learning models. J Thorac Dis.

[24] Saadatfar H, Khosravi S, Joloudari JH, Mosavi A, Shamshirband S (2020). A new K-nearest neighbors classifier for big data based on efficient data pruning. Mathematics.

[25] Nusinovici S, Tham YC, Chak Yan MY, Wei Ting DS, Li J, Sabanayagam C (2020). Logistic regression was as good as machine learning for predicting major chronic diseases. J Clin Epidemiol.

[26] Shamshirband S, Fathi M, Dehzangi A, Chronopoulos AT, Alinejad-Rokny H (2020). A Review on deep learning approaches in healthcare systems: taxonomies, challenges, and open issues. J Biomed Informat..

[27] Ashish L, Sravan KV, Yeligeti S. Ischemic heart disease detection using support vector machine and extreme gradient boosting method. Mater Today Proc 2021(6).

[28] Kumar B, Gupta D (2021). Universum based Lagrangian twin bounded support vector machine to classify EEG signals. Comput Meth Programs Biomed..

[29] Vapnik V, Vapnik V (1995). The natural of statistical learning theory. Technometrics.

[30] Veropoulos K, Campbell C, Cristianini N. Controlling the sensitivity of support vector machines. In: Proceedings of the international joint conferences on artificial intelligence. 1999.

[31] Kang Q, Shi L, Zhou M, Wang X, Wu Q, Wei Z (2018). A distance-based weighted undersampling scheme for support vector machines and its application to imbalanced classification. IEEE Trans Neural Netw Learn Syst..

[32] Hazarika BB, Gupta D, Applications. Density-weighted support vector machines for binary class imbalance learning. Neural Comput. 2020(2).

[33] Anaissi A, Goyal M, Catchpoole DR, Braytee A, Kennedy PJ (2016). Ensemble feature learning of genomic data using support vector machine. PLoS ONE.

[34] Pouriyeh S, Vahid S, Sannino G, Pietro GD, Gutierrez JB. A comprehensive investigation and comparison of machine learning techniques in the domain of heart disease. In: 22nd IEEE symposium on computers and communication (ISCC 2017): workshops—ICTS4eHealth; 2017.

[35] Huang HF, Liu J, Zhu Q, Wang RP, Hu GS (2014). A new hierarchical method for inter-patient heartbeat classification using random projections and RR intervals. Biomed Eng Online.

[36] Shorewala V. Early detection of coronary heart disease using ensemble techniques. Informat Med Unlocked. 2021;26.

[37] Alsafi HES, Ocan ON. A novel intelligent machine learning system for coronary heart disease diagnosis. Appl Nanosci. 2021.

[38] Aghaei A, Mohraz M, Shamshirband S. Effects of media, interpersonal communication and religious attitudes on HIV-related stigma in Tehran, Iran. Inform Med Unlocked. 2020;18.

[39] Joloudari JH, Joloudari EH, Saadatfar H, Ghasemigol M, Razavi SM, Mosavi A (2020). Coronary artery disease diagnosis; ranking the significant features using a random trees model. Int J Environ Res Public Health.

[40] Liu H, Zhou M, Liu Q (2019). An embedded feature selection method for imbalanced data classification. IEEE/CAA J Autom Sin.

[41] Singh BK (2019). Determining relevant biomarkers for prediction of breast cancer using anthropometric and clinical features: a comparative investigation in machine learning paradigm. Biocybern Biomed Eng Online.

[42] Ma L, Fu T, Blaschke T, Li M, Tiede D, Zhou Z (2017). Evaluation of feature selection methods for object-based land cover mapping of unmanned aerial vehicle imagery using random forest and support vector machine classifiers. Isprs Int J Geo-Inf..

[43] Wang H, Khoshgoftaar TM, Gao K. A comparative study of filter-based feature ranking techniques. In: 2010 IEEE international conference on information reuse & integration; 2010 4–6 Aug 2010.

[44] Plackett RL (1983). Karl Pearson and the chi-squared test. Int Stat Rev.

[45] Abdar M, Kalhori SRN, Sutikno T, Subroto IMI, Arji G (2015). Comparing performance of data mining algorithms in prediction heart diseases. Int J Electr Comput Eng.

[46] Ali H, Mohd Salleh MNB, Saedudin R, Hussain K, Mushtaq MF. Imbalance class problems in data mining: a review. Indon J Electr Eng Comput Sci. 2019;14(3).

[47] Weiss GM (2004). Mining with rarity—problems and solutions: a unifying framework. Acm Sigkdd Explor Newsl.

[48] Sun B, Chen HY, Wang JD, Xie H (2018). Evolutionary under-sampling based bagging ensemble method for imbalanced data classification. Front Comput Sci.

[49] Gu X, Ni T, Wang H (2014). New fuzzy support vector machine for the class imbalance problem in medical datasets classification. TheScientificWorldJOURNAL.

[50] Velusamy D, Ramasamy K (2021). Ensemble of heterogeneous classifiers for diagnosis and prediction of coronary artery disease with reduced feature subset. Comput Meth Programs Biomed.

[51] Chen XF, Li XM, Chen XB, Huang XM. Analysis of emergency misdiagnosis of 22 cases of aortic dissection. Clin Misdiagn Misther. 2016;29(1).

[52] Harris RJ, Kim S, Lohr J, Towey S, Velichkovich Z, Kabachenko T (2019). Classification of aortic dissection and rupture on post-contrast CT images using a convolutional neural network. J Digit Imaging.

[53] Wu J, Qiu J, Xie E, Jiang W, Zhao R, Qiu J (2019). Predicting in-hospital rupture of type A aortic dissection using random forest. J Thorac Dis.

[54] Teng Y, Gao Y, Feng SX (2012). Diagnosis and misdiagnosis analysis of 131 cases of aortic dissection. Chin J Misdiagn.

[55] Wang HY, Zhu ZY (2016). Analysis on clinical features and misdiagnosis of 58 patients with acute aortic dissection. Hainan Med J.

